# Application of the extended parallel process model and risk perception attitude framework to obesity knowledge and obesity prevention behaviors among Korean adults

**DOI:** 10.1186/s12889-024-18268-5

**Published:** 2024-03-08

**Authors:** Jong-Ho Park, Su-Jung Nam, Jeong-Eun Kim, Nam-Chul Kim

**Affiliations:** 1 365MC Obesity Clinic, Suwon, South Korea; 2 Department of Consumer Sciences, Convergence Program for Social Innovation, College of Social Sciences, Sungkyunkwan University, 25-2, Sungkyunkwan-Ro, JongnoGu, Seoul, South Korea; 3 365MC Hospital, Seoul, South Korea; 4 365MC Hospital, Busan, South Korea

**Keywords:** Extended parallel process model, Risk perception attitude, Obesity knowledge, Obesity prevention behavior

## Abstract

**Background:**

Perceiving oneself as obese has been associated with weight loss attempts. However, such a perception may not sufficiently drive significant weight reduction in many individuals. Hence, relying solely on the traditionally emphasized perceived risk of behavioral changes in obesity is challenging. This study used an extended parallel process model and a risk perception attitude framework to explore the influence of perceived risk and perceived efficacy on individual obesity knowledge and obesity prevention behaviors.

**Methods:**

Data were obtained from 1,100 Korean adults aged 40–69 years through an online survey conducted in October 2022. Multinomial logistic regression and analysis of variance were employed to assess the relationships among perceived risk, perceived efficacy, obesity knowledge, and obesity prevention behaviors.

**Results:**

Sex was associated with being underweight, overweight, and obese. Moreover, perceived severity was associated with obesity, whereas perceived susceptibility was associated with overweight and obese. Response efficacy was related to being overweight alone, whereas self-efficacy was associated with being underweight, overweight, and obese. The main effects of sex and perceived risk, and their interaction effect were statistically significant for obesity knowledge. Additionally, the main effects of sex, perceived risk, and perceived efficacy on obesity prevention behaviors were statistically significant.

**Conclusions:**

The extended parallel process model and risk perception attitude framework proved effective in classifying obesity based on body mass index, obesity knowledge, and obesity prevention behaviors.

## Background

By 2035, the number of individuals with overweight and obesity worldwide could surpass 4 billion, ultimately exceeding the 2.6 billion record set in 2020 [1]. This development signifies a notable increase from 38% of the global population by 2020 to over 50% by 2035. Moreover, the prevalence of obesity alone is expected to increase from 14 to 24% within the same timeframe, affecting nearly 2 billion adults, children, and adolescents by 2035 [1]. Obesity is associated with a multitude of morbidities, including heart disease, diabetes, hypertension, and certain cancers [2, 3], and the World Health Organization (WHO) has defined obesity as a disease (International Classification of Diseases, Tenth Revision [ICD-10]: E66.0–E66.9).

The intricate nature of motivating health behavioral change is apparent through the multitude of conceptual frameworks that have been postulated [4, 5]. Among these, the health belief model (HBM) [6], protection motivation theory (PMT) [7], and parallel process model (PPM) [8] underscore the significance of risk perception as the driving force behind the adoption of behaviors aimed at mitigating risks.

However, a considerable number of individuals do not perceive obesity as a serious concern [9, 10] and remain unaware of its associated health risks [11]. Moreover, although perceiving oneself as overweight has been linked to weight loss attempts, this perception appears to be insufficient for driving weight reduction among many individuals [12, 13]. Therefore, regarding obesity, expecting behavioral changes based solely on the traditionally emphasized perceived risk is challenging. Furthermore, recent studies have suggested that risk perception alone cannot fully explain all the factors that motivate individuals to take preventive actions. Yoon and Seo [14] proposed that individuals’ perceptions of risk do not always align with the actual level of risk and have a greater influence on the decision-making process than the objective risk itself. Consequently, individuals may opt for different behaviors even when facing equal health risks. Understanding the underlying factors that shape behavioral changes is crucial for explaining such behavioral variations. Accordingly, the extended parallel process model (EPPM) [15] and risk perception attitude (RPA) framework [16] incorporate perceived efficacy, recognizing that relying solely on risk perception is inadequate for predicting individuals’ behavioral changes.

The current study used EPPM and RPA to explore the influence of perceived efficacy and perceived risk on individual obesity knowledge and obesity prevention behaviors. The inclusion of perceived efficacy and risk was aimed at enhancing our understanding of the factors that influence individuals’ engagement in efforts to prevent obesity.

The research questions (RQs) investigated in this study were as follows;

- RQ1. How do the factors (i.e., perceived severity, perceived susceptibility, response efficacy, and self-efficacy) of the EPPM, obesity knowledge, and obesity prevention behaviors relate to groups classified by body mass index (BMI)?
- RQ2. How are obesity groups distributed based on RPA?
- RQ3. How do obesity knowledge and obesity prevention behaviors vary by RPA type?

### Extended parallel process model (EPPM)

The EPPM is a theoretical framework that elucidates individuals’ behavioral responses to fear-provoking messages [15]. Originally derived from the PPM proposed by Leventhal [8] and centered on the interplay between danger and fear control, the EPPM delves into the mechanisms through which individuals engage in adaptive protective behaviors as a result of their attempts to mitigate potential risks. Furthermore, EPPM incorporates key elements of the PMT proposed by Roger [7]. The EPPM contends that individuals do not solely engage in preventive behaviors by perceiving the presence of danger; rather, they require a robust conviction regarding the efficacy of disease prevention. Perceived risk serves as a driving force for the adoption of protective behaviors to ward off disease, whereas the nature of active or passive behaviors is mediated by individuals’ perceived efficacy of an action or behavior [15].

According to the EPPM, when individuals encounter fear-inducing stimuli, they engage in two simultaneous modes of message processing: cognitive processing (perceived efficacy appraisal) and emotional processing (perceived risk appraisal) [15]. The EPPM conceptualizes the process of evaluating risk as a means of fear control, with perceived risk playing a crucial role in determining this process. Additionally, the evaluation of coping mechanisms is depicted as a risk control process, with perceived efficacy serving as the key determinant. These two factors are further subdivided into the following dimensions: (1) perceived severity and susceptibility in relation to perceived risk, and (2) response efficacy and self-efficacy in relation to perceived efficacy. Perceived severity refers to an individual’s perception of the magnitude of risk. Perceived susceptibility refers to an individual’s perception of the likelihood of risk affecting them. Response efficacy refers to an individual’s perception that an action, if carried out, can successfully control risk. Self-efficacy refers to an individual’s perception of their competence in performing the tasks needed to control risk.

The EPPM differs from previous models (e.g., HBM, PMT, and PPM) that focus on fear appeal. While previous fear appeal models primarily concentrated on why fear appeals succeeded through perceived risk, the EPPM also explores why fear appeals fail through the interaction between perceived risk and perceived efficacy. The EPPM posits that when an individual experiences fear and perceives a high level of risk, risk and fear control processes are triggered based on the level of perceived efficacy. If individuals perceive high risk and efficacy, they are likely to exhibit an adaptive response; that is, a risk-control response. Conversely, if an individual perceives high risk but low efficacy, they tend to display a maladaptive response; that is, a fear-control response [17]. Hence, perceived efficacy plays a crucial moderating role in the risk- and fear-control processes.

### Risk perception attitude (RPA)

The RPA framework developed by Rimal and Real [16] aims to categorize individuals into groups based on their perceived risk and efficacy concerning diseases. The RPA framework draws on the PMT by Rogers [7] and the EPPM introduced by Witte [15]. Additionally, the RPA framework seeks to elucidate variations in health outcomes observed among different groups [16, 18].

According to the RPA framework proposed by Rimal and Juon [19], individuals can be categorized into four distinct groups based on their perceived risk and efficacy. First, the responsive group (RG) is postulated to exhibit higher levels of health behaviors than the other groups, given its high perceived risk and efficacy. The avoidance group (AG) faces a dilemma in which their motivation to undertake preventive behaviors is undermined by high perceived risk and low perceived efficacy. Consequently, their fear levels may intensify, resulting in avoidant behaviors. For the proactive group (PG) and indifferent group (IG), the motivation to take action against potential hazards is low owing to their low perceived risk; in this case, perceived efficacy cannot easily stimulate health behaviors [16]. Thus, the RPA model assumes that, despite disparities in the magnitude of perceived efficacy between the PG and IG, these groups will not show discernible disparities in health behavioral outcomes owing to their low perceived risk [16].

The RPA model offers a theoretical framework that enables the classification of target audiences and the development of tailored campaign strategies for each group. The effectiveness of the RPA framework has been substantiated in various health domains including cardiovascular health [18], skin cancer prevention [16, 20], lung cancer prevention [21], human immunodeficiency virus /human immunodeficiency virus (AIDS/HIV) prevention [22], and coronavirus disease 2019 (COVID-19) prevention [23].

## Methods

### Participants

This study analyzed data acquired from Macromill Embrain (https://embrain.com), a market research enterprise in Seoul, South Korea. The survey was administered online in October 2022. The participants were selected from Embrain’s extensive panel database, encompassing over 1 million participants. Specifically, this study focused on individuals aged 40–69 years who were residing in Korea during the study period. Individuals who were retired or classified as younger adults and did not meet the criteria for receiving preventive medical services under the universal healthcare system were excluded from this study. These selection criteria facilitated the establishment of a cohort characterized by homogeneous health behaviors, consequently enhancing the precision of the ensuing outcomes. The potential participants were approached online and written informed consent was obtained from each participant before their inclusion. Ethical approval was obtained from the Jeonju University Research Ethics Committee (JJIRB-220,526-HR-20,220,501).

### Participant characteristics

Table 1 shows the demographic characteristics of the participants. Age distribution revealed that 34.5%, 32.7%, and 32.7% of the individuals belonged to the 40–49, 50–59, and 61–69-year age groups, respectively, with an equal representation of males and females. The largest segment of participants consisted of college graduates. Moreover, most respondents were married, with 47.3% employed full-time, and their income levels showing a uniform distribution. Regarding self-rated health, 50.5% of the respondents reported that their health was fair. According to the obesity criteria based on the Korea Disease Control and Prevention Agency (KDCA), individuals are categorized as underweight, normal weight, overweight, and obese if their BMI is < 18.5 kg/m^2^, 18.5–22.9 kg/m^2^, 23.0–24.9 kg/m^2^, and ≥ 25 kg/m^2^, respectively. Based on the KDCA obesity criteria, 38 (3.5%), 506 (46.0%), 248 (22.6%), and 307 (27.9%) participants in this study were categorized as underweight, healthy weight, overweight, and obese, respectively.

**Table 1:** Participants’ characteristics

Category		Frequency	%
Sex			
	Male	540	49
	Female	560	51
Age			
	40–49	380	35
	50–59	360	33
	60–69	360	33
Education			
	Elementary school graduate	4	0.4
	Middle school graduate	11	1
	High school graduate	284	26
	College graduate	693	63
	Graduate school or higher	108	9.8
Work type			
	Self-employed	105	9.5
	Full-time worker	520	47
	Part-time worker	105	9.5
	Housewife	219	20
	Inoccupation	40	3.6
	Retirement	88	8
	Other	23	2.1
Marital status			
	Yes	937	97
	No	163	3.5
Monthly income^a^			
	≤ 100	100	9.1
	101–200	149	14
	201–300	221	20
	301–400	204	19
	401–500	148	14
	501–600	106	9.6
	601–700	63	5.7
	700≤	109	9.9
Self-rated health			
	Very poor	12	1.1
	Poor	163	15
	Average	555	51
	Good	360	33
	Very good	10	0.9
Classification according to BMI			
	Underweight	38	3.5
	Healthy	506	46
	Overweight	248	23
	Obese	307	28

*Note*: ^a^South Korean KRW 10,000 (USD 1 = KRW 1,019.20)

### Measures

Table 2 shows specific questions about perceived risk (i.e., perceived severity and perceived susceptibility), perceived efficacy (i.e., response efficacy and self-efficacy), obesity knowledge, and obesity prevention behaviors.

Assessments for perceived risk and efficacy were conducted using the EPPM through the implementation of a five-point Likert scale [20, 22]. Consistent with prior research [24, 25] that employed the RPA framework to categorize participants based on median values, this study employed the same approach to classify individuals based on their medians of risk perception and perceived efficacy. The median values for perceived risk and perceived efficacy were both 3.50. Consequently, individuals in the high perceived risk and high perceived efficacy categories were designated as the RG. Those with a low perceived risk and high perceived efficacy were classified as having PG. Those with a high perceived risk and low perceived efficacy were classified as having AG. Those with low perceived risk and low perceived efficacy were referred to as the IG group.

Obesity knowledge was calculated as the sum of correct responses minus the sum of incorrect answers. The respondents were asked to answer five true-or-false questions regarding obesity-related facts according to the Korean Ministry of Health and Welfare [26]. The respondents indicated whether each statement was true or false or if they were unsure. Correct answers were scored one point, uncertain responses were scored zero points, and a point was subtracted from the total score for incorrect answers. The total possible score ranged from − 5 (all incorrect answers) to 5 (all correct answers). Obesity prevention behavior***s*** were measured using four items obtained from the Obesity Prevention Scale developed by the Korean Ministry of Health [26]. The participants evaluated their engagement with each preventive measure on a five-point Likert scale.

**Table 2:** Measurement of perceived risk, perceived efficacy, obesity knowledge, and obesity prevention behaviors

Items	Mean (SD)	Cronbach’s α
***Perceived severity***		0.744
SE1. Obesity is a fatal disease	3.48 (0.88)	
SE2. Obesity is a painful disease	2.72 (1.02)	
SE3. I would be disappointed and shocked if I were obese	3.26 (0.923)	
SE4. I think that the time and economic losses caused by obesity are substantial	3.62 (0.87)	
***Perceived susceptibility***		0.824
SU1. Anyone can be obese	3.79 (0.83)	
SU2. My family can be obese	3.59(0.56)	
SU3. The people around me, including my friends and colleagues, can be obese	3.80 (0.71)	
***Response efficacy***		0.722
RE1. Prevention should be a top priority to avoid obesity	4.08 (0.67)	
RE2. I think preventive behaviors against obesity are effective	4.14 (0.67)	
RE3. Preventing obesity is necessary	3.67 (0.84)	
***Self-efficacy***		0.800
SE1. I can take good care of myself to avoid becoming obese	3.47 (0.87)	
SE2. I can thoroughly engage in preventive behaviors to avoid becoming obese	3.46 (0.88)	
SE3. I will undergo a thorough examination to prevent obesity	3.32 (0.87)	
***Obesity Prevention Behavior***		0.751
B1. I maintain a balanced diet by incorporating a variety of foods.	3.15 (0.82)	
B2. I avoid consuming salty foods.	3.32 (0.86)	
B3. I walk or engage in exercise at least five times a week, with each session lasting at least 30 min	3.66 (1.14)	
B4. I make an effort to sustain a healthy weight.	3.34 (0.97)	
	Correct Response	
***Obesity Knowledge***		
K1. Consuming food rapidly is associated with an increased risk of obesity.	Truth	
K2. Addressing obesity may elevate the risk of developing osteoporosis.	Truth	
K3. Weight gain as you age is a natural and inherent aspect of the aging process.	Truth	
K4. Genetics is not a determining factor in obesity.	False	
K5. Consuming fried foods is associated with weight gain.	Truth	

### Analysis

Stata SE18 was used to conduct the multinomial logistic regression. The main objective of this study was to investigate the influence of participants’ characteristics, perceived risk, perceived efficacy, obesity knowledge, and obesity prevention behavior on the classification of groups based on BMI. Moreover, an analysis of variance (ANOVA) was performed to examine the main and interaction effects of perceived risk and perceived efficacy on obesity knowledge and obesity prevention behavior.

## Results

### Multinomial logistic regression results of participants’ characteristics, perceived risk, and perceived efficacy on classification according to BMI

The results for RQ 1 aligned with the findings of the multinomial logistic regression analysis presented in Table 3. First, compared with the healthy weight group, the likelihood of being underweight increased among females, those who were economically inactive, and those with increased self-efficacy. Second, compared with the healthy weight group, the probability of belonging to the overweight group increased among males and those with increased perceived susceptibility, increased response efficacy, decreased self-efficacy, increased obesity knowledge, and decreased obesity prevention behavior. Finally, compared with the healthy weight group, the probability of belonging to the obesity group increased among males; married individuals; and those with decreased perceived severity, increased perceived susceptibility, decreased self-efficacy, increased obesity knowledge, and decreased obesity prevention behavior.

**Table 3:** Multinomial logistic regression of characteristics, perceived risk, and perceived efficacy on classification according to BMI

	Underweight				Overweight				Obesity			
	B	Odds ratio	95%CI	*p*	B	Odds ratio	95%CI	*p*	Coefficient	Odds ratio	95%CI	*p*
**Characteristic**												
Female	1.404	4.072	1.411 11.754	0.009	-1.230	0.292	0.200 0.428	0.000	-1.512	0.220	0.149 0.326	0.000
Age	0.001	1.001	0.955 1.050	0.953	0.004	1.004	0.982 1.028	0.707	0.016	1.017	0.994 1.041	0.152
Education: Under. Highschool	−.435	0.647	0.275 1.521	0.318	0.209	1.233	0.843 1.804	0.281	−.157	0.855	0.574 1.272	0.439
Non-Marriage	0.355	1.426	0.523 3.888	0.488	−.330	0.719	0.444 1.165	0.181	−.939	0.391	0.234 0.653	0.000
Economic inactivity^a^	0.808	2.243	1.035 4.681	0.041	0.058	1.059	0.724 1.549	0.767	0.051	1.052	0.713 1.553	0.798
Income: Less than 401	−.300	0.741	0.343 1.599	0.444	−.232	0.792	0.553 1.135	0.204	−.062	0.939	0.650 1.358	0.738
Self-rated health	−.141	0.868	0.512 1.472	0.600	0.064	1.067	0.831 1.369	0.612	−.056	0.945	0.734 1.216	0.659
**Perceived risk**												
Perceived severity	−.311	0.733	0.472 1.137	0.166	0.063	1.066	0.854 1.331	0.574	−.294	0.745	0.593 0.936	0.012
Perceived susceptibility	−.167	0.846	0.525 1.363	0.492	0.244	1.276	1.001 1.627	0.049	0.558	1.748	1.348 2.266	0.000
**Perceived Efficacy**												
Response efficacy	0.012	1.012	0.554 1.848	0.969	0.412	1.511	1.139 2.004	0.004	0.172	1.188	0.885 1.595	0.251
Self-efficacy	0.844	2.326	1.229 4.404	0.010	−.519	0.595	0.444 0.797	0.000	-1.014	0.363	0.268 0.490	0.000
**Obesity Knowledge**	−.043	0.958	0.784 1.169	0.671	0.132	1.142	1.037	0.007	0.100	1.106	1.003 1.220	0.044
**Obesity prevention behaviors**	−.145	0.865	0.436 1.717	0.679	−.345	0.708	0.519 0.965	0.029	−.682	0.506	0.369 0.692	0.000

^a^The categories of housewife, inoccupation, retirement, and others are those who are not currently engaged in economic activities and earning monetary income; Reference group = Healthy weight; Cox and Snell = 0.273, -2 Log likelihood = 2211.523; BMI, body mass index

### Classification according to perceived risk and perceived efficacy

The results for RQ2 are presented in Table 4. A total of 182 respondents (16.5%) were categorized into the IG, which is characterized by low perceived risk and low perceived efficacy. The PG comprised 247 respondents (22.5%) with a low perceived risk and high perceived efficacy. The AG group comprised 301 individuals (27.4%) with high perceived risk and low perceived efficacy. Finally, the RG included 370 participants (33.6%) with high perceived risk and high perceived efficacy. The proportion of RG respondents was relatively high. The χ^2^ value for the group classification, 1.494, was not statistically significant.

**Table 4:** Groups based on perceived risk and perceived efficacy

		Perceived efficacy		χ^2^
		Low	High	
Perceived risk	Low	Indifferent group 182 (16.5%)	Proactive group 247 (22.5%)	1.494
	High	Avoidance group 301 (27.4%)	Responsive group 370 (33.6%)	

### Obesity knowledge and obesity prevention behavior according to groups based on risk perception attitude

The results for RQ3 correspond to the findings obtained from the GLM analysis presented in Table 5. This table presents the results of the main and interaction effects of perceived risk and perceived efficacy on obesity knowledge and obesity prevention behavior, considering the influence of sex, which consistently affects underweight, overweight, and obese relative to healthy weight.

**Table 5:** Knowledge and behaviors according to groups based on risk perception attitude framework

Category	Obesity knowledge							Obesity prevention behaviors					
	M (S.E.)	95%CI	SS	F	*p*	η_p_^2^		M (S.E.)	95%CI	SS	F	*p*	*η*_p_^2^
Sex			57.423	20.096	0.000	0.018				4.805	13.439	0.000	0.012
Male	1.501 (0.074)	1.356 1.647						3.271 (0.026)	3.220 3.323				
Female	1.975 (0.075)	1.827 2.122						3.408 (0.027)	3.356 3.461				
Perceived risk			24.607	8.612	0.003	0.008				1.735	4.851	0.028	0.004
High	1.893 (0.066)	1.764 2.022						3.299 (0.023)	3.253 3.344				
Low	1.583 (0.083)	1.420 1.745						3.381 (0.029)	3.324 3.439				
Perceived efficacy			3.683	1.289	0.256	0.001				83.837	234.46	0.000	0.177
High	1.678 (0.069)	1.542 1.814						3.626 (0.025)	3.578 3.674				
Low	1.798 (0.080)	1.642 1.954						3.054 (0.028)	2.999 3.109				
Perceived risk$\times$sex			14.933	5.226	0.022	0.005				0.258	0.722	0.396	0.000
Perceived efficacy$\times$sex			3.821	1.337	0.248	0.001				0.012	0.034	0.853	0.000
Perceived risk$\times$perceived efficacy			3669	0.234	0.629	0.000				0.126	0.351	0.554	0.000
R^2^			0.0307							0.2004			
Root MSE			1.6904							0.5979			

**Fig. 1:**
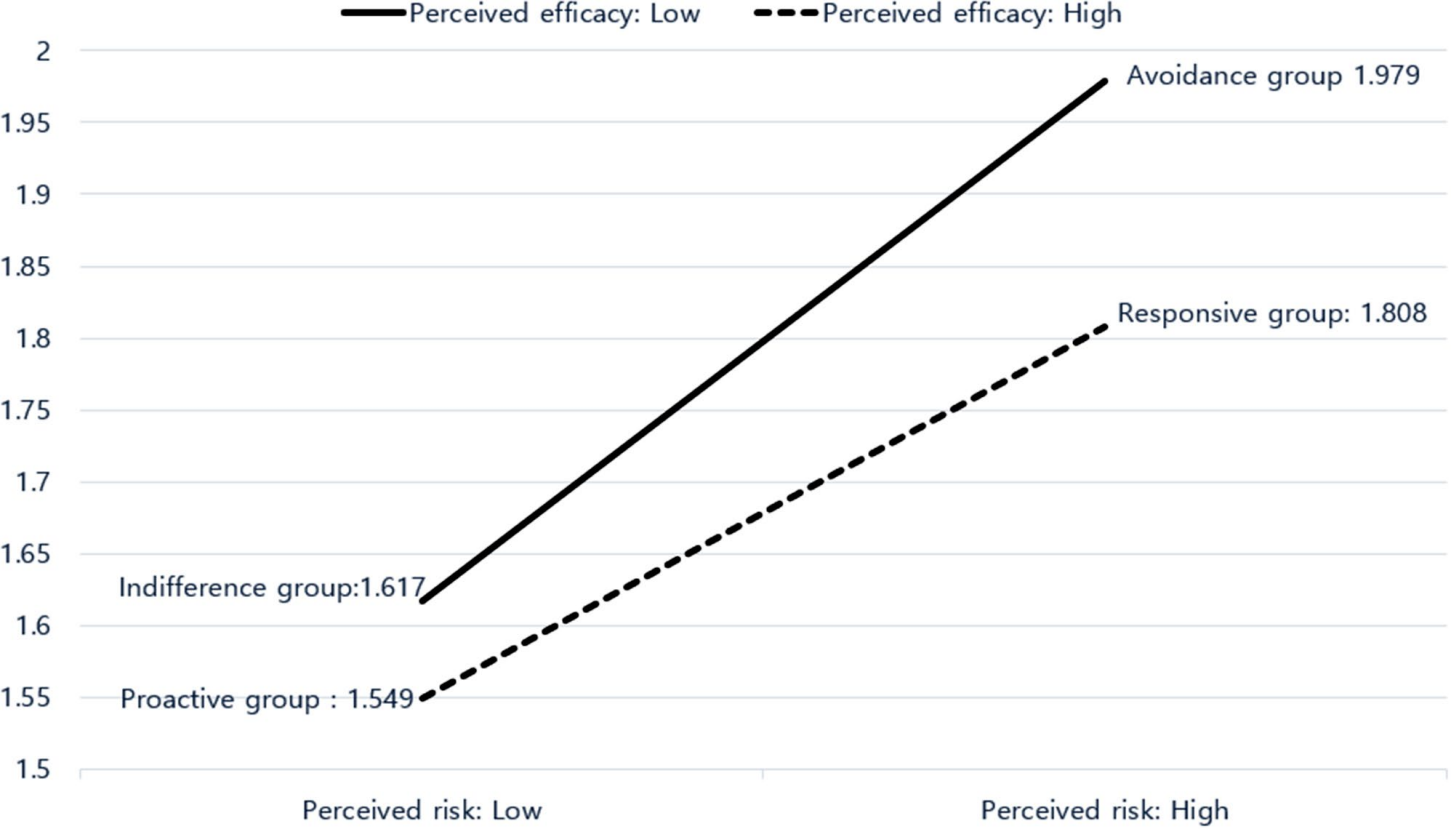
Obesity knowledge according to perceived risk and perceived efficacy

The main effects of sex and the perceived risk of obesity on knowledge were statistically significant. As illustrated in Fig. 1, participants in AG (1.979) and RG (1.808), characterized by higher perceived risk, demonstrated higher knowledge scores than those in IG (1.617) and PG (1.549), characterized by lower perceived risk.

**Fig. 2:**
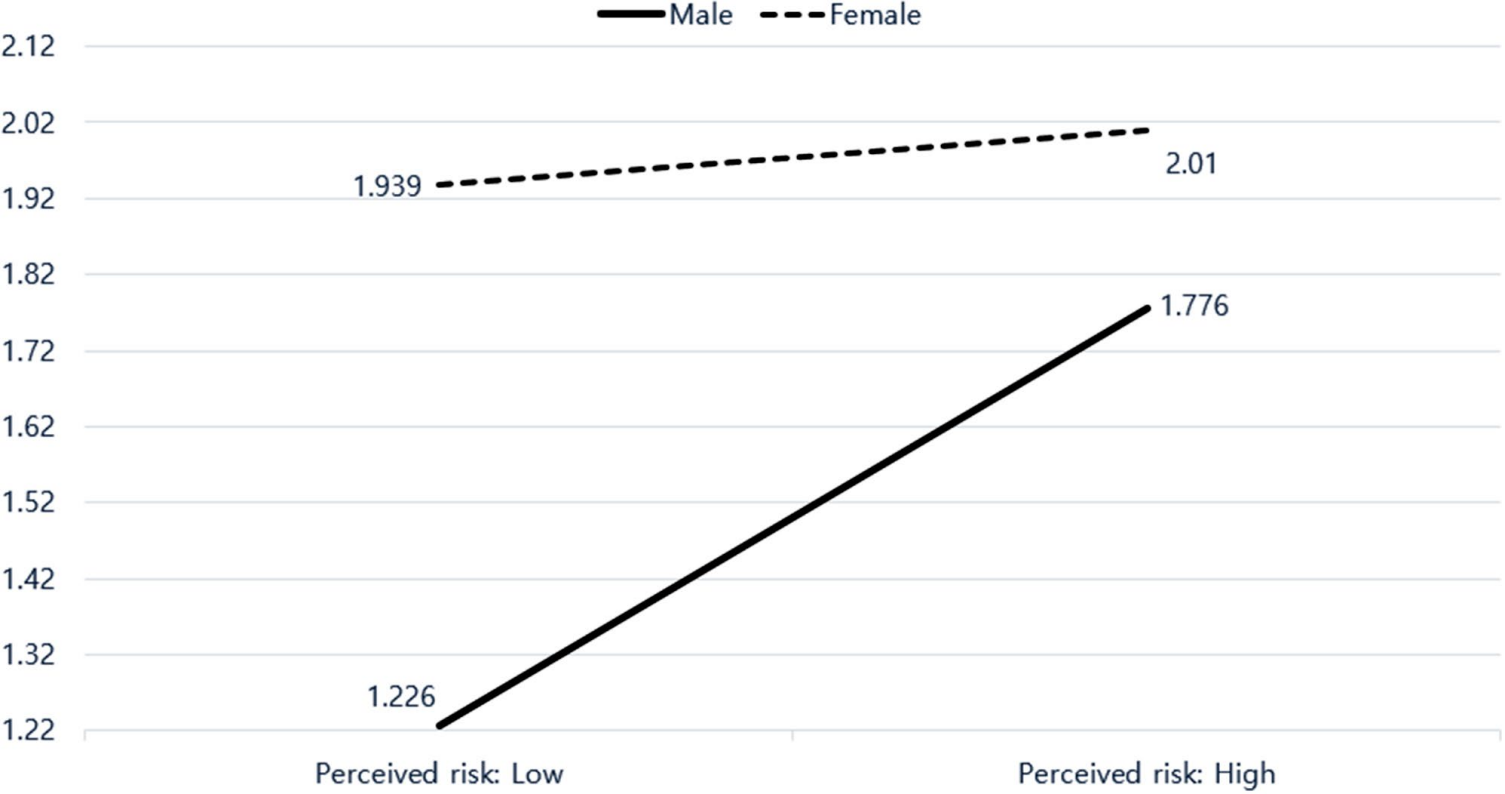
Obesity knowledge according to sex and perceived risk

Furthermore, the interaction effect between sex and perceived risk of obesity knowledge was statistically significant (Fig. 2).

Additionally, Table 6 shows the simple main effects of sex and perceived risk. The difference in sex within the low perceived risk group and the difference in perceived risk among men were statistically significant.

**Table 6:** Simple main effects of sex and perceived efficacy on obesity knowledge

	SS	F	p	η_p_^2^
Sex @ low perceived risk	53.718	18.799	0.000	0.017
Sex @ high perceived risk	9.076	3.176	0.075	0.003
Perceived risk @ male	39.529	13.834	0.000	0.012
Perceived risk @ female	0.637	0.223	0.637	0.000

**Fig. 3:**
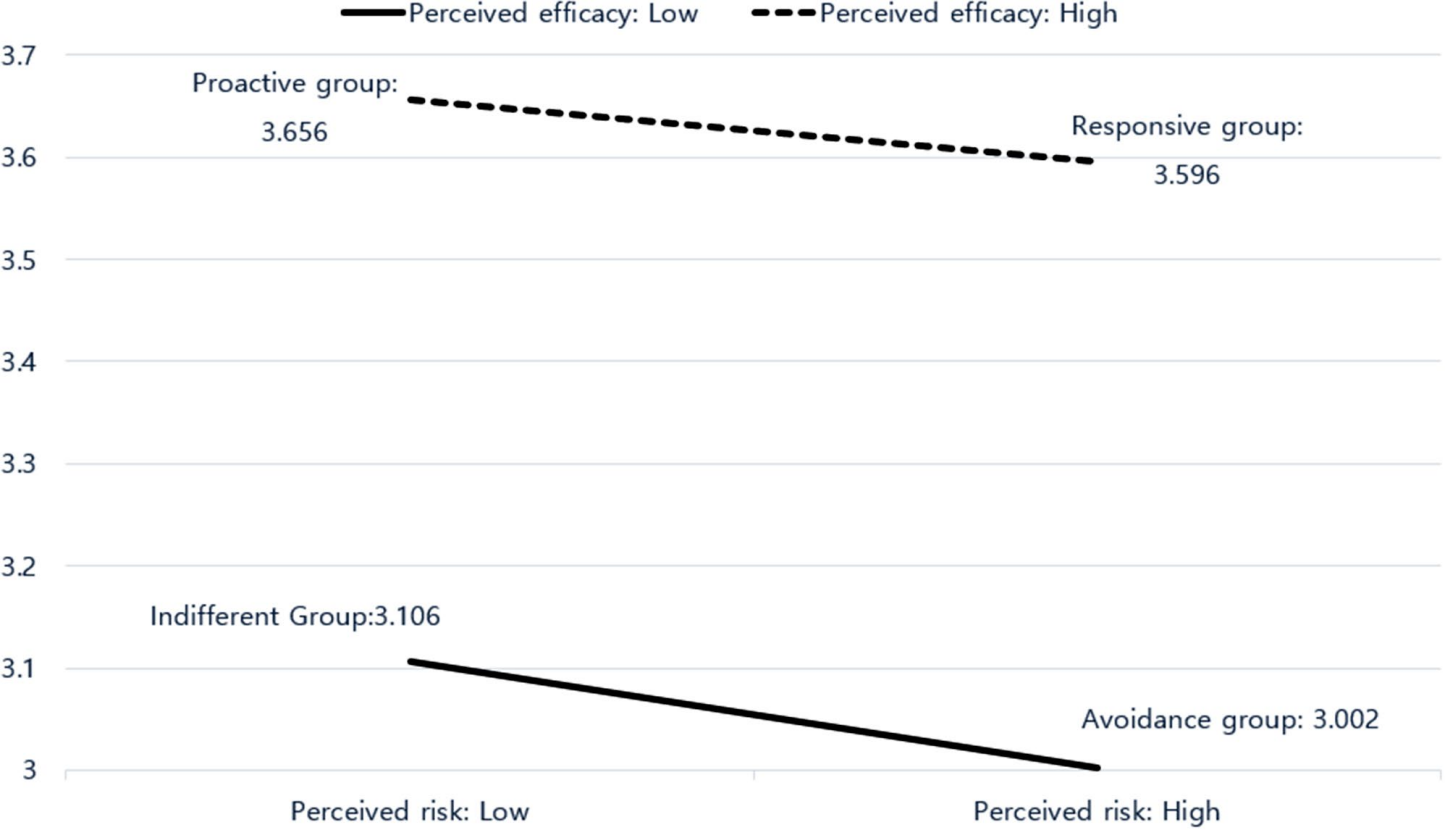
Obesity prevention behavior according to perceived risk and perceived efficacy

The main effects of sex, perceived risk, and perceived efficacy on obesity prevention behaviors were statistically significant; however, the interaction effects were not. As shown in Fig. 3, participants in the PG (3.656) and RG (3.596) with higher perceived efficacy showed higher behavioral scores than those in the IG (3.106) and AG (3.002) with lower self-efficacy.

## Discussion

This study analyzed a survey of individuals aged 40–69 years to examine the applicability of the EPPM and RPA for classification according to BMI, obesity knowledge, and obesity prevention behaviors. The results confirmed the influences of perceived risk and efficacy, which are the main components of the EPPM and RPA, on the aforementioned factors.

The factors influencing the likelihood of belonging to the underweight, overweight, and obese groups relative to the healthy weight group were sex, perceived risk, perceived efficacy, obesity knowledge, and obesity prevention behaviors.

Previous studies have reported differences in the prevalence of obesity between males and females [27–30]. Furthermore, reports have indicated that substantial differences in the prevalence of overweight and obesity and negative mental health resulting from being overweight or obese are more common among females than among males [31–34]. Therefore, the influence of sex must be considered in research on overweight and obesity.

Regarding perceived risk, perceived severity affected only the obese group, whereas perceived susceptibility affected the overweight and obese groups. The lower the perceived severity, the more probable it is that individuals will be classified into the obese group rather than the healthy weight group. Conversely, greater perceived susceptibility increases the likelihood of individuals belonging to the overweight or obese groups rather than to the group with a healthy weight. Interestingly, individuals in the obese group tended to underestimate the physical and economic risks associated with obesity. Moreover, this group and those around them had a high likelihood of developing obesity. This observation highlights the distinct effects of perceived severity and susceptibility, which are two components of perceived risk that manifest in contrasting ways. However, despite this, perceived severity and susceptibility still appear to be integrated as factors of perceived risk, and there seems to be no need to interpret them as independent factors within the framework of the EPPM in the context of obesity. Nevertheless, it is more valid to consider perceived severity as a factor that diminishes perceived risk in the obese group, while perceived susceptibility is a factor that heightens perceived risk in the overweight and obese groups.

Concerning perceived efficacy, response efficacy only influenced the overweight group, while self-efficacy exerted an impact not only on the overweight and obese groups but also on the underweight group. Specifically, in the overweight group, a significantly high evaluation of response efficacy was observed concerning obesity prevention behaviors. Conversely, obesity was not significantly associated with response efficacy. Consequently, distinct approaches should be implemented to educate overweight and obese individuals on obesity prevention behaviors. Specifically, the response efficacy in individuals with obesity must be enhanced.

Self-efficacy was identified as the most influential factor among the EPPM components. Higher self-efficacy scores were associated with an increased likelihood of belonging to the underweight group, whereas lower self-efficacy scores were associated with a higher probability of belonging to the overweight or obese groups. Consequently, the enhancement of self-efficacy, particularly in overweight and obese individuals, must be prioritized to promote the adoption of obesity prevention behaviors. Furthermore, the impact of obesity knowledge and prevention behaviors on BMI classification was not substantiated in the underweight group. In the overweight and obese groups, the influence was statistically significant. These findings indicate that higher obesity knowledge increased the likelihood of belonging to the overweight and obese groups. These results also suggest that the overweight and obese groups displayed relatively high levels of obesity knowledge, possibly because of their heightened awareness and involvement in obesity. Moreover, fewer obesity prevention behaviors increased the likelihood of belonging to the overweight and obese groups. When respondents were classified according to perceived risk and perceived efficacy relative to obesity, the RG showed the highest proportion (33.6%), followed by the IG (16.5%). The main effects of sex and perceived risk and the interaction effect of perceived risk and sex were statistically significant relative to obesity knowledge. Additionally, the main effects of sex, perceived risk, and perceived efficacy on obesity prevention behaviors were statistically significant.

Initially, female respondents demonstrated a greater degree of obesity knowledge than their male counterparts. Individuals with high perceived risk exhibited higher levels of obesity knowledge than those with low perceived risk. However, contrary to prior research findings [32–34], the AG displayed higher levels of obesity knowledge than the RG in this study. Nevertheless, the statistical analysis revealed non-significant outcomes for the main and interaction effects of perceived efficacy on obesity knowledge. These results imply that the disparity between the AG and the RG was not statistically significant.

Finally, regarding obesity prevention behaviors, the group with a low perceived risk achieved higher scores than the group with a high perceived risk. This finding contradicts those reported previously [16, 18, 22, 23], wherein higher perceived risk was associated with greater behavioral changes. The EPPM and RPA posit that a high level of perceived risk is necessary to elicit behavioral changes. In cases where the perceived risk is not sufficiently high, individuals are likely to exhibit non-responsive behaviors, avoidance, or a tendency to disengage from preventive actions. Nonetheless, in the context of obesity, in contrast to other illnesses or risks, the perceived severity of the risk may be considerably low. Despite WHO’s acknowledgment of obesity as a disease, individuals often tend to underestimate its severity [9–11]. Consequently, in the context of obesity, the influence of perceived risk may be diminished compared with that of other illnesses or risks. Therefore, a cautious approach is warranted when considering the impact of perceived risk in the application of EPPM and RPA to address obesity-related behaviors. Conversely, the effect size (*η*_*p*_^*2*^) for the impact of perceived efficacy was substantial at 0.177, indicating the significant and sizable effect of perceived efficacy on obesity prevention behaviors [35]. Ultimately, the results of the present study revealed that EPPM and RPA provided explanatory power for classification according to BMI, obesity knowledge, and obesity prevention behaviors, with perceived efficacy exerting a stronger influence than perceived risk. This study introduces a novel research paradigm by applying EPPM and RPA, which have been previously employed in diverse health and risk contexts, to obesity. However, this study has several limitations, as outlined below.

First, this study deviates from previous research by uncovering contrasting findings regarding obesity prevention behaviors in relation to perceived risk. Specifically, the results indicated that individuals with a lower perceived risk exhibited higher scores on obesity prevention behavior. Hence, follow-up studies are required to validate and substantiate the effects of perceived risk.

Second, the inclusion criteria restricted the age range of the participants to individuals aged 40–69 years, thereby excluding children and adolescents. Future research should involve these populations as they are crucial to include in comprehensive obesity studies.

## Data Availability

The data are available upon request. Please contact the corresponding authors for further details.

## Abbreviations

WHO: World Health Organization
HBM: Health Belief Model
PMT: Protection Motivation Theory
PPM: Parallel Process Model
EPPM: Extended Parallel Process Model
RPA: Risk Perception Attitude
BMI: Body Mass Index
IG: Indifferent Group
PG: Proactive Group
AG: Avoidance Group
RG: Responsive Group
KDCA: Korea Disease Control and Prevention Agency
